# Structural Characterization and Anti-Inflammatory Properties of an Alginate Extracted from the Brown Seaweed *Ericaria amentacea*

**DOI:** 10.3390/md24010041

**Published:** 2026-01-13

**Authors:** Maha Moussa, Serena Mirata, Lisa Moni, Valentina Asnaghi, Marina Alloisio, Simone Pettineo, Maila Castellano, Silvia Vicini, Mariachiara Chiantore, Sonia Scarfì

**Affiliations:** 1 Department of Earth, Environment and Life Sciences, University of Genoa, 16132 Genoa, Italy; maha.moussa@edu.unige.it (M.M.); serenamira94@gmail.com (S.M.); valentina.asnaghi@unige.it (V.A.); mariachiara.chiantore@unige.it (M.C.); 2 National Biodiversity Future Center (NBFC), 90133 Palermo, Italy; 3 Inter-University Centre for the Promotion of the 3Rs Principles in Teaching & Research, 10129 Turin, Italy; 4 Department of Chemistry and Industrial Chemistry (DCCI), University of Genoa, 16146 Genoa, Italy; lisa.moni@unige.it (L.M.); marina.alloisio@unige.it (M.A.); pettineo.simone@gmail.com (S.P.); maila.castellano@unige.it (M.C.); silvia.vicini@unige.it (S.V.)

**Keywords:** brown algae, *Ericaria amentacea*, sodium alginate, biological effects, gene expression

## Abstract

Brown algae of the Cystoseira genus are recognized as valuable sources of bioactive compounds, including polysaccharides. Within the framework of current restoration efforts regarding damaged *Ericaria amentacea* populations in the Mediterranean Sea, the valorization of apices derived from ex situ cultivation waste represents a sustainable opportunity for industrial and biomedical applications. In this study, sodium alginate (SA) was extracted from *E. amentacea* apex by-products using a hydrothermal–alkaline method and subsequently chemically characterized. FTIR analysis showed O-H, C-H, and COO- stretching compatible with commercial alginates, while 1H-NMR spectroscopy indicated high β-D-mannuronic acid content, with an M/G ratio of 2.33. The extracted SA displayed a molecular weight of 1 × 10^4^ g/mol and a polydispersity index of 3.5. The bioactive properties of the SA extract were investigated in chemico and in vitro. SA exhibited remarkable antioxidant activity, showing significant DPPH and nitric oxide-radical-scavenging capacity. Furthermore, SA demonstrated a strong anti-inflammatory effect in LPS-stimulated macrophages through modulation of several inflammatory mediators (i.e., IL-6, IL-8/CXCL5, MCP-1, and TNF-α). In particular, SA promoted a striking iNOS gene expression inhibition, which, paired with its direct NO-scavenging ability, paves the way for future pharmacological use of *E. amentacea* derivatives, particularly if sustainably obtained from restoration activity waste.

## 1. Introduction

Macroalgae are widely recognized as a valuable source of bioactive compounds with applications in the food, cosmeceutical, and pharmaceutical industries, as well as in the production of phycocolloids [1,2]. According to the Food and Agriculture Organization (FAO) of the United Nations, global seaweed production reached approximately 34.7 million tons in 2019. Of this total, red and brown seaweed represent the majority, contributing about 53% and 47%, respectively, while green seaweed accounts for only 0.054% [3]. Brown seaweed, in particular, is regarded as a sustainable and renewable biomass source due to its widespread availability and fast growth [4]. Moreover, the bioprocessing of seaweed is considered environmentally friendly, as it is biodegradable and biocompatible, making it suitable for various industrial and biomedical applications [5].

Phycocolloids are polysaccharides associated with the cell walls and intercellular spaces of certain seaweed species [6]. A typical example is alginate, a naturally occurring polysaccharide, first identified by British chemist E.C.C. Stanford in 1881 [7]. It represents the primary structural polysaccharide in brown seaweed, and it is typically found in the cell wall and matrix as a mixture of cationic salts naturally present in seawater. In its native state, alginate occurs as a mixture of alginic calcium, magnesium, and sodium salts, contributing both to the strength and the flexibility of the algal tissue [8].

At the molecular level, alginates are anionic, linear binary copolymers composed of β-D-mannuronic acid (M) and α-L-guluronic acid (G) residues, linked through (1→4) glycosidic bonds. The stereochemical difference between mannuronic and guluronic acid arises from the configuration at the C-5 carbon in the uronic acid ring. These two acids are arranged into three distinct block types: homopolymeric sequences of mannuronic acid (MM-blocks), homopolymeric sequences of guluronic acid (GG-blocks), and heteropolymeric sequences of alternating M and G units (MG-blocks) [9,10]. Mannuronic acid units form β-(1→4) bonds, resulting in linear and flexible M-block segments. In contrast, guluronic acid units are connected by α-(1→4) bonds, introducing steric hindrance around the carboxyl groups, which leads to folded, rigid conformations in the G-block segments [10]. The ratio of mannuronic to guluronic acid (M/G ratio) varies depending on the algal species, the specific tissue, and even the harvesting season [11]. These structural characteristics, particularly the M/G ratio, the block arrangement, and the distribution of M and G units, have a significant impact on the physicochemical properties of alginates. To date, approximately 200 distinct alginate types have been identified, each with varying M/G ratios depending on the source and extraction conditions [12]. The water-soluble form of sodium alginate (SA) is especially valued in the food and pharmaceutical industries due to its ability to form hydrogels in the presence of divalent cations such as Ca^2+^ [13]. In such cases, guluronic acid residues from different polymer chains interact with calcium ions via their carboxylic groups (COO^−^), forming a thermally irreversible, water-insoluble three-dimensional network commonly referred to as the “egg-box” model [14]. Owing to their biocompatibility, non-toxicity, biodegradability, and polyelectrolytic nature, alginates are particularly suitable for various biological and biomedical purposes, including anti-inflammatory and antioxidant applications [15].

Inflammation is a fundamental component of the innate immune response, playing a crucial role in protecting the body against pathogenic invasion and tissue injury. Traditionally, it is characterized by vasodilation and leukocyte infiltration, manifesting clinically as erythema, heat, edema, and pain [16]. From an evolutionary standpoint, the inflammatory cascade is an essential defense mechanism designed to eliminate harmful stimuli and initiate tissue repair through a transient, self-regulated process [17]. However, when inflammatory triggers persist and/or the inflammatory response fails to resolve, the process can become chronic. In contrast to acute inflammation, which is typically short-lived and beneficial, chronic inflammation is often subclinical and prolonged, progressively contributing to cellular dysfunction and tissue damage. Furthermore, chronic inflammation is nowadays a broadly recognized feature of several diseases such as diabetes, hypertension, atherosclerosis, and cancer [18]. To counteract inflammation, various synthetic anti-inflammatory agents have been developed. While effective, many of these compounds are associated with adverse side effects. As a result, there is growing interest in the exploration of naturally derived bioactive compounds (i.e., polysaccharides) with dual anti-inflammatory and antioxidant capabilities.

A key molecular mechanism linking inflammation and cellular damage is oxidative stress, which arises from the overproduction of reactive oxygen species (ROS). Reactive oxygen species (ROS)—including superoxide anion (•O_2_^−^), hydrogen peroxide (H_2_O_2_), organic peroxide (ROOR′), hydroxyl radical (•OH), and peroxynitrite (ONOO^−^)—are natural byproducts of cellular metabolism and of exposure to xenobiotics [19,20]. Although ROS play essential roles in intracellular signaling and certain physiological processes, excessive ROS production results in oxidative stress, leading to cellular and molecular damage. Elevated intracellular ROS levels can damage organic macromolecules such as proteins [21], DNA [22], and lipids [23]. When oxidative damage exceeds the cellular antioxidant defense capacity, it impairs key cellular functions and ultimately triggers cell death via apoptosis or necrosis [24].

In the Mediterranean Sea, macroalgal forests are predominantly composed of species belonging to *Cystoseira sensu lato* (s.l.) (Fucales, Phaeophyceae). In the last few decades, these forests have experienced significant decline or local extinction due to anthropogenic pressures and the impacts of climate change [25]. One of the species impacted by this decline is *Ericaria amentacea*, a Mediterranean endemic fucoid alga that colonizes the lower intertidal zone, forming dense monospecific belts. To mitigate its decline, various ex situ restoration activities have been implemented to support the recovery of *E. amentacea* populations along the Mediterranean and Ligurian coasts [26,27]. These restoration efforts generate biological waste, i.e., apical segments of the algal fronds, used to obtain the gametes needed for the cultivation process [28]. In this study, we aimed to extract and characterize, for the first time, sodium alginate polymers from *E. amentacea* using the apical waste material generated during ex situ cultivation for restoration purposes. Furthermore, we investigated the antioxidant and anti-inflammatory properties of these SA extracts by means of in chemico and in vitro cellular tests, showing very promising biological properties.

## 2. Results

### 2.1. Chemical Composition

The chemical content of *Ericaria amentacea* sodium alginate (SA) is presented in Table 1. Colorimetric assays revealed that the SA extract contained 44.25% total sugar and 49% uronic acids. In addition, 0.52% proteins and 0.93% polyphenols were detected.

### 2.2. Chemical Analyses

#### 2.2.1. Fourier-Transform Infrared Spectroscopy

**Figure 1:**
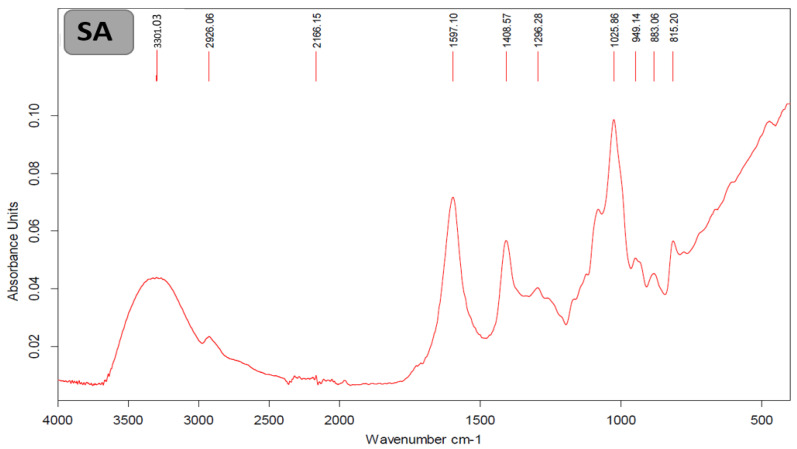
FTIR spectrum of *E. amentacea* SA.

The Fourier-transform infrared (FTIR) spectrum of SA is shown in Figure 1. The chemical structure of this SA exhibited O-H, C-H, and COO- stretching, which are represented by bands at 3301 cm^−1^, 2926 cm^−1^, and 1597–1408 cm^−1^, respectively, according to commercial alginates [29].

#### 2.2.2. NMR Spectroscopy

**Figure 2:**
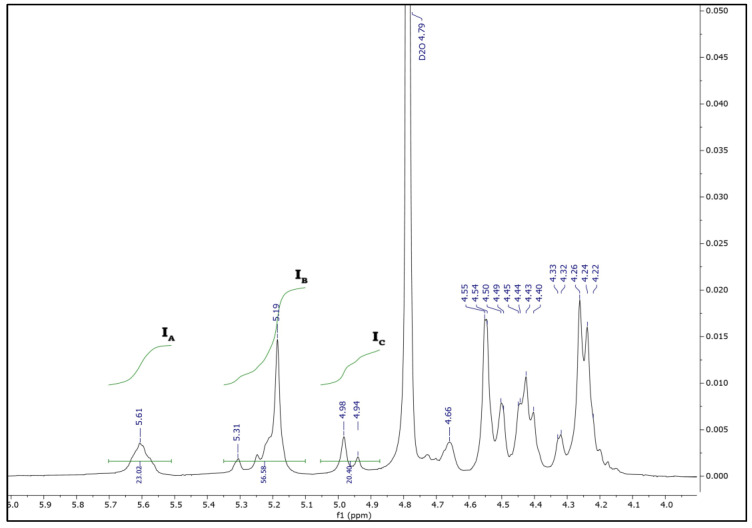
^1^H NMR analysis of SA extracted from *E. amentacea* by setting the residual HOD signal in D_2_O to 4.79 ppm: signal I_A_ = guluronic acid anomeric proton (G-1), signal I_B_ = overlap between the mannuronic acid anomeric proton (M-1) and the H-5 of alternating blocks (GM-5), signal I_C_ = guluronic acid H-5 position (block GG-5G).

The characterization of the chemical composition and block distribution of the SA extracted from *E. amentacea* was performed by ^1^H NMR spectroscopy. The acquired data showed a typical 400 MHZ-^1^H NMR spectrum (Figure 2) with a set of specific signals characteristic of the sodium alginate fraction, confirming the high purity of the extracted polysaccharide. The signal assignment was achieved by comparison with previously reported chemical shifts [30,31].

The spectrum revealed signals of the guluronic acid anomeric proton (G-1) at 5.61 ppm (signal I_A_) and guluronic acid H-5 (GG-5G) at 4.98 ppm (signal I_C_), while the overlap between the mannuronic acid anomeric proton (M-1) and the C-5 of alternating blocks (GM-5) was identified at 5.19 ppm (signal I_B_).

The area of the described signals (I_A_, I_B_, and I_C_) was calculated and used to define the M/G ratio, the molar pair frequencies of the monads of mannuronic and guluronic acid (F_M_ and F_G_), and the diad sequences (F_GG_, F_MM_, F_MG_, or F_GM_). Indeed, the proportions of each block (F_G_ and F_M_) and the homogeneous (F_GG_ and F_MM_) and heterogeneous (F_GM_ and F_MG_) blocks of SA extracted from *E. amentacea* were estimated by applying the commonly used equations of Grasdalen et al. [30], summarized as follows:

F_G_ = I_A_/(I_B_ + I_C_)

F_GG_ = I_C_/(I_B_ + I_C_)

F_M_ = 1 − F_G_

F_GM_ = F_G_ − F_GG_

F_MM_ = F_M_ − F_MG_

M/G = F_M_/F_G_

The chemical composition of SA extracted from *E. amentacea*, as summarized in Table 2, was determined using integral ^1^H NMR values, as well as the equations mentioned above.

### 2.3. Molecular Weight Determination

Intrinsic viscosity analysis was performed to evaluate the molecular weight of the SA extract. As shown in Table 3, SA was characterized by a weight average molecular mass (M_w_) of 1 × 10^4^ g/mol, a number average molecular mass (M_n_) of 2.9 × 10^3^ g/mol, and a polydispersity index q (M_w_/M_n_) of 3.5.

### 2.4. Biological Properties

#### 2.4.1. Antioxidant Potential DPPH Radical-Scavenging Activity

**Figure 3:**
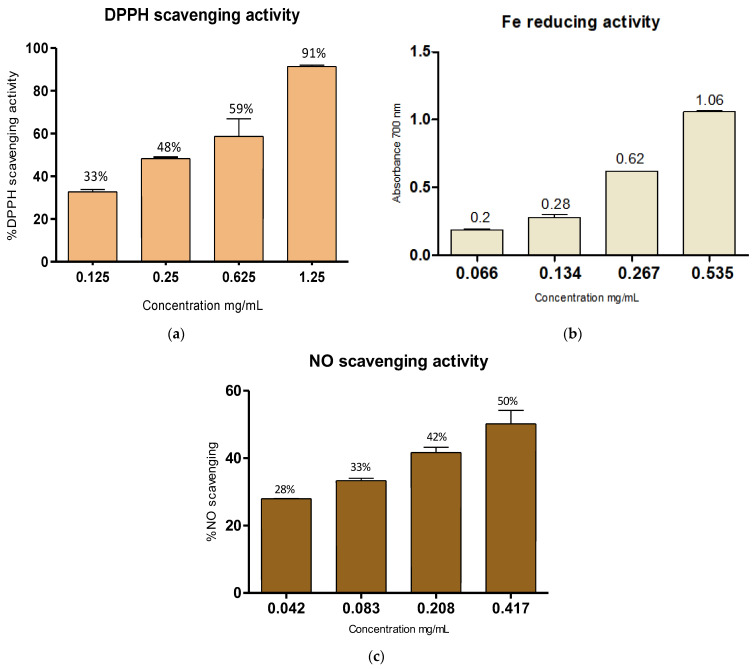
The antioxidant activity of *E. amentacea* SA in spectrophotometric tests. (**a**) ROS scavenging activity by the DPPH assay; (**b**) Fe reducing activity of *E. amentacea* SA; (**c**) NO-scavenging potential of *E. amentacea* SA. Ascorbic acid (AA) and sodium nitroprusside (SN) were used as positive controls, AA in the DPPH and Fe reducing activity assay, SN in the NO assay. Data are the mean ± S.D. of three experiments performed in duplicate.

The overall radical-scavenging activity of the SA measured by the DPPH assay revealed an elevated antioxidant potential (Figure 3a). At 1.25 mg/mL and 0.625 mg/mL tested concentrations, in fact, the SA showed a scavenging potential higher than 50%, with 91% and 59% reduction capacities, respectively, and a resulting IC_50_ of 0.410 mg/mL.

- Reducing Fe (III) Power Assay

SA’s ability to reduce the Fe^3+^/ferricyanide complex to the ferrous form was evaluated by following the potassium ferricyanide method (Figure 3b). The reducing power of SA increased with increasing concentrations (from 0.067 to 0.535 mg/mL), obtaining A_700_ values of 1.06 A.U. at 0.535 mg/mL, corresponding to the highest SA concentration used.

- NO-Scavenging Activity

The NO-scavenging activity of the SA extract was evaluated by the nitroprusside NO donor assay. The reducing power of SA proved to be dose-dependent (from 0.042 to 0.417 mg/mL). The NO IC_50_ scavenging potential of the SA extract was 0.417 mg/mL (Figure 3c).

#### 2.4.2. Anti-Inflammatory Potential

- Cytotoxicity test

**Figure 4:**
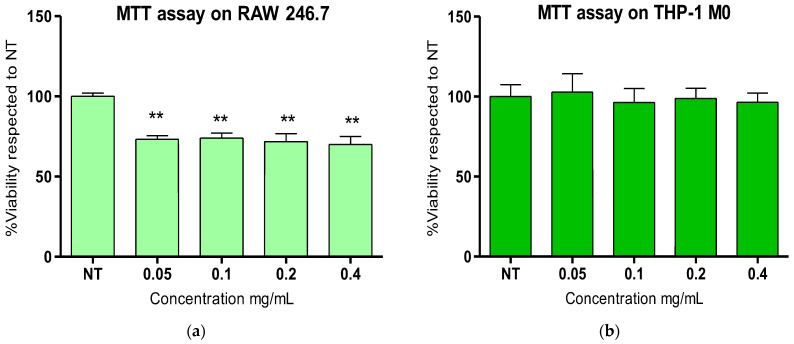
MTT assay for the assessment of in vitro cytotoxicity. (**a**) In vitro cytotoxicity of SA at various (from 0.05 to 0.4 mg/m/L) concentrations evaluated on RAW 264.7 murine macrophages. (**b**) In vitro cytotoxicity of SA at the same concentrations evaluated on human differentiated THP-1 M0 macrophages. The results are expressed as a percentage of viability with respect to the control (untreated cells) and are the mean ± SD of three experiments performed in five replicates. ANOVA was significant (i.e., *p* < 0.05) in (**a**), with ** *p* < 0.01 vs. NT in Tukey post-test.

The cytotoxicity of SA at various concentrations was evaluated in murine RAW 264.7 macrophages and in human THP-1-derived M0 macrophages to investigate the safe use of these extracts for human health applications. The RAW 264.7 cells and differentiated THP-1 M0 cells were incubated for 24 h with various dilutions of SA (from 0.05 to 0.4 mg/mL), and then, the cell viability was evaluated by the MTT test and compared to untreated control cells (Figure 4). Concerning RAW 264.7 murine macrophages, a slight decrease in the cell number (around 30%) for all tested concentrations was observed after SA exposure (Figure 4a) as compared to control cells, probably due to a slower growth rate in the presence of the SA, which was concentration-independent. Conversely, for the differentiated human THP-1 M0 macrophages, the results showed no significant effects on cell growth for all tested concentrations (0.05 and 0.4 mg/mL, Figure 4b). These data indicate that *E. amentacea* SA might be safely used in humans due to the absence of significant toxicity.

- Gene expression analysis for LPS-activated RAW 264.7 macrophages

RAW 264.7 cells were stimulated with 100 ng/mL of pro-inflammatory bacterial endotoxin (LPS). The expression of inflammatory cytokines and mediators, such as interleukin 1β (IL-1β); interleukin 6 (IL-6); the murine homologue of human IL-8 cytokine, i.e., C-X-C Motif Chemokine Ligand 5 (CXCL5); tumor necrosis factor (TNF-α); and cyclooxygenase-2 (COX-2), was evaluated by qPCR to assess the in vitro anti-inflammatory potential of the *E. amentacea* SA at 0.2 and 0.4 mg/mL concentrations.

**Figure 5:**
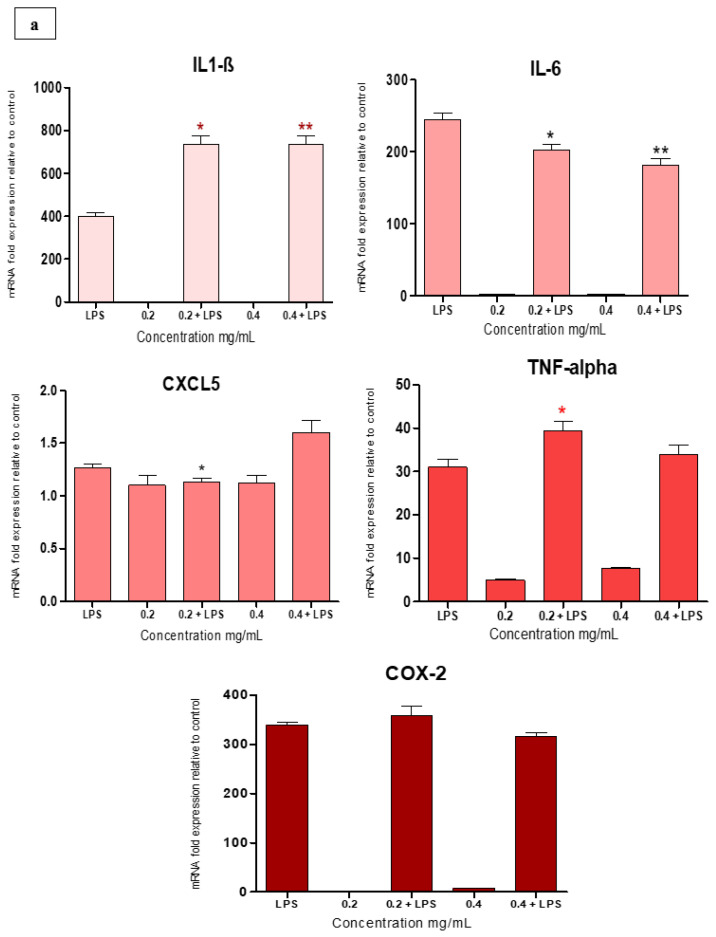

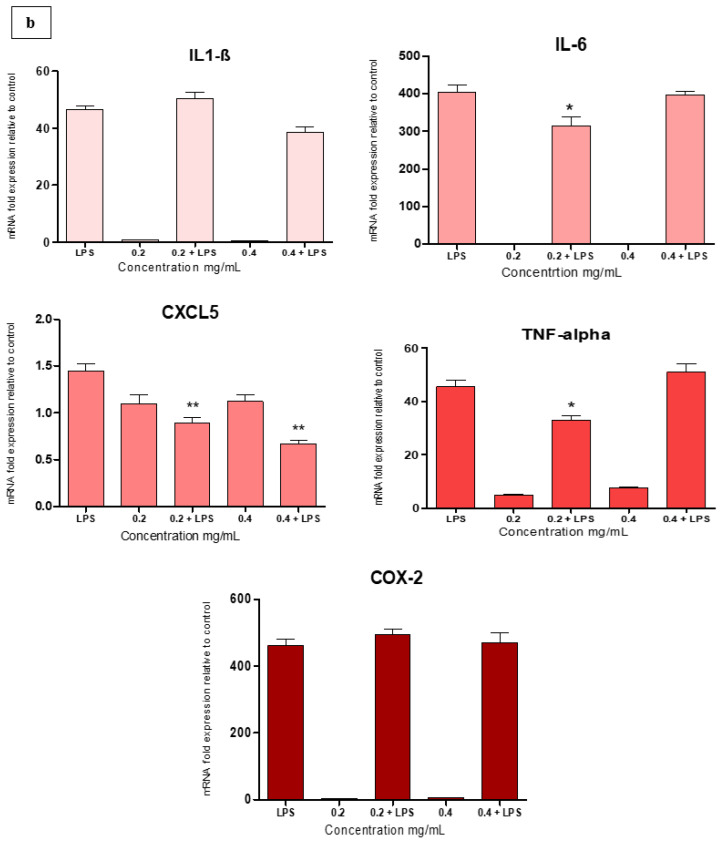
*E. amentacea* SA inhibition of gene expression in LPS-activated RAW 264.7 macrophages. Gene expression measured by qPCR analysis of IL-1β, IL-6, CXCL5, TNF-α, and COX-2, after incubation for 8 h (**a**) and 24 h (**b**), with or without LPS (100 ng/mL), and in the presence or absence of 0.2 and 0.4 mg/mL SA. Data are normalized on the GAPDH housekeeping gene and expressed as mRNA fold increase compared to control, untreated cells. Results are the mean ± SD of three experiments performed in duplicate. ANOVA was significant in each histogram (*p* < 0.05), with * *p* < 0.05, and ** *p* < 0.01 vs. LPS in Tukey post-test.

All investigated cytokines and inflammatory mediators were significantly overexpressed in murine macrophages following 8 or 24 h of LPS treatment (Figure 5a,b), as compared to untreated controls. After 8 h of LPS stimulation, the SA at the lowest concentration (0.2 mg/mL) exhibited inhibition potentials of 17% and 12% on the overexpression of IL-6 and CXCL5, respectively, as compared to LPS alone (Figure 5a). On the other hand, the highest SA concentration (0.4 mg/mL) led to the inhibition of the IL-6 gene by 26%. Instead, a significant gene expression increase in IL-1β, exceeding that of LPS alone, was detected after both SA treatments, while no significant effects were detected in either SA treatment on the COX-2 and TNF-α gene expression profiles after the 8 h LPS stimulation.

After 24 h, the lowest SA concentration (0.2 mg/mL) led to a significant inhibition of IL-6, TNF-α, and CXCL5 overexpression, as compared to LPS alone, by 25%, 39%, and 20%, respectively (Figure 5b). On the other hand, at the highest SA concentration (0.4 mg/mL), we observed the inhibition of IL-1β and CXCL5 overexpression, as compared to LPS alone, by 15% and 40%, respectively. No inhibitory effects were detected in the induced COX-2 gene overexpression by either SA concentration. In conclusion, a mild but significant inhibition of IL-6, CXCL5, and TNF-α gene expression was achieved in inflamed murine macrophages following *E. amentacea* SA treatment. Parallel experiments were performed in RAW 264.7 cells using 0. 4 mg/mL of commercial low and medium molecular weight SA (CL and CM, respectively) and are reported in Figure S1. In this case, at 8 h (panel A) in the presence of LPS, both CL and CM significantly stimulated IL-1β, IL-6, and CXCL5 expression as compared to LPS alone, with no significant effect on TNF-α and COX-2 stimulation. This trend was also maintained at 24 h (panel B), with the only difference observed in TNF-α being slight inhibition caused by both alginates as compared to LPS alone. These data reveal significantly different behaviors between the *E. amentacea*-extracted SA and the two commercially available SAs, with the former showing potential, although mild, anti-inflammatory activity in activated murine macrophages not paralleled by the latter.

- Gene expression analysis of LPS-activated THP-1 M0

Differentiated human THP-1 M0 macrophages were stimulated with 100 ng/mL of LPS. The expression of inflammatory cytokines, i.e., IL-1β, IL-6, IL-8, monocyte chemoattractant protein-1 (MCP-1), and TNF-α, was evaluated by qPCR to assess the anti-inflammatory potential of the *E. amentacea* SA at a 0.4 mg/mL concentration.

**Figure 6:**
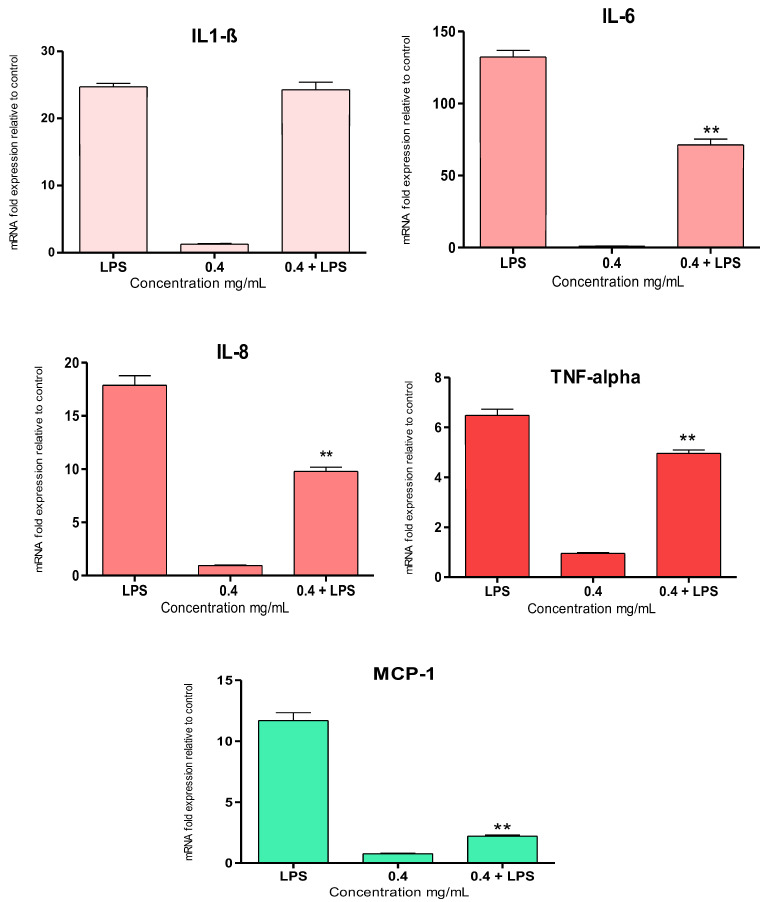
*E. amentacea* SA inhibition of gene expression in THP-1 M0 macrophages. Gene expression measured by qPCR analysis of IL-1β, IL-6, IL-8, TNF-α, and MCP-1 after THP-1 M0 macrophage incubation, with or without LPS (100 ng/mL), and in the presence or absence of 0.4 mg/mL SA for 24 h. Data are normalized on the HPRT-1 housekeeping gene and expressed as mRNA fold increase compared to control, untreated cells. Results are the mean ± SD of three experiments performed in duplicate. ANOVA was significant in each histogram (*p* < 0.05), with ** *p* < 0.01 vs. LPS in Tukey post-test.

All inflammatory cytokines were significantly upregulated after 24 h of LPS incubation (Figure 6), and SA treatment resulted in the significant inhibition of IL-6, IL-8, MCP-1, and TNF-α, as compared to LPS alone, by 46%, 46%, 81%, and 23%, respectively. Similarly to the results obtained from RAW 264.7 macrophages, also in THP-1 M0 cells, no inhibitory effects were detected in the LPS-stimulated IL-1β gene overexpression. Also in this case, parallel experiments were performed on THP-1 M0 macrophages by using 0.4 mg/mL of commercial CL and CM SA (Figure S2), and the results showed very different behavior with respect to the *E. amentacea* SA extract. In particular, no inhibitory effect on LPS-overexpressed cytokines was observed in the presence of CL SA, except for a light decrease in MCP-1, while a slight but significant inhibition of all cytokines was observed in the case of the CM treatment, as compared to LPS alone, indicating a mild anti-inflammatory effect on activated human macrophages caused by the medium molecular weight SA.

- NO-Scavenging activity and iNOS gene expression in LPS-Activated RAW 264.7 Macrophages

**Figure 7:**
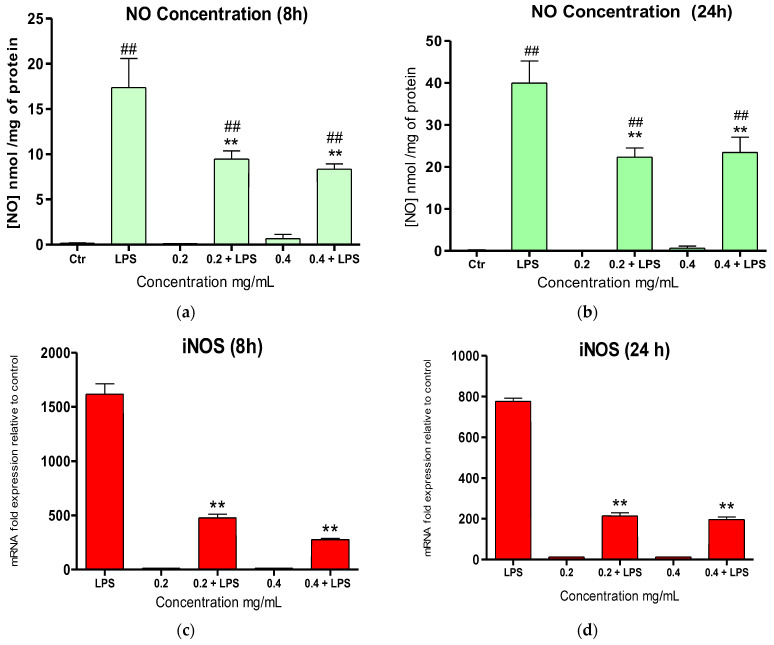
NO-scavenging activity and iNOS gene expression in cellular assay. (**a**,**b**) Intracellular NO production measured by Griess assay in RAW 264.7 murine macrophages incubated for 8 h (**a**) and 24 h (**b**) with LPS (100 ng/mL) in the presence or absence of 0.2 and 0.4 mg/mL of SA. The results are expressed as a percentage of NO production, with respect to control, untreated cells, and are the mean ± SD of two assays performed in triplicate. (**c**,**d**) Gene expression measured by qPCR analysis of iNOS after RAW 264.7 cell incubation, for 8 h (**c**) and 24 h (**d**), with or without 100 ng/mL LPS and in the presence or absence of 0.2 and 0.4 mg/mL SA. Data are normalized on the GAPDH housekeeping gene and expressed as mRNA fold increases compared to control, untreated cells. Results are the mean ± SD of three experiments performed in duplicate. ANOVA was significant in each histogram (*p* < 0.05), with ** *p* < 0.01 vs. LPS in Tukey post-test and ^##^ *p* < 0.01 vs. negative control in Tukey post-test.

RAW 264.7 macrophages were stimulated with LPS (100 ng/mL), and the NO production was evaluated after incubation in the presence or absence of the *E. amentacea* SA at 0.2 and 0.4 mg/mL (Figure 7). LPS stimulation led to significant NO overproduction in RAW 264.7 macrophages (17.35 and 40.01 nmol/mL/mg protein after 8 and 24 h incubation; Figure 7a and b, respectively) compared to control cells, in which the production was undetectable. After 8 h of incubation, NO production was significantly inhibited (by 45.5% and 52%) by both the 0.2 and 0.4 mg/mL SA concentrations, respectively. Similarly, the LPS-stimulated NO overproduction was significantly inhibited by the two SA concentrations after 24 h of incubation by 44.7% and 34%, respectively.

In relation to this important inhibition of NO production at the biochemical level, we also aimed to quantify the enzyme responsible for its production, the inducible NO synthase (iNOS). The gene expression level was quantified after LPS stimulation in the presence and absence of the *E. amentacea* SA (Figure 7c,d). Indeed, a strong impairment of inducible NO synthase expression was achieved in inflamed murine macrophages following the SA treatment. In detail, after 8 h, we observed inhibitions of 70% and 83% on the overexpression of the iNOS gene, as compared to LPS alone (Figure 7c), in the presence of 0.2 and 0.4 mg/mL SA, respectively. Following this trend, also after 24 h, both SA concentrations maintained a significant 70% inhibition of iNOS overexpression, as compared to LPS alone (Figure 7d). Parallel experiments were performed using 0.4 mg/mL of commercial CL and CM SA in the presence of LPS, also in this case measuring NO release and iNOS gene expression in RAW 264.7 macrophages. The results are shown in Figure S3. In detail, concerning NO release, no inhibition of this molecular species was observed after the 8 h treatment in the presence of LPS and CL, while a significant increase was observed in the presence of LPS and CM SA as compared to LPS alone. Conversely, at 24 h, the two commercial SAs caused a slight decrease in NO production as compared to LPS alone. Furthermore, concerning the iNOS gene expression, at both 8 and 24 h, the two commercial SAs caused a significant decrease, as compared to overexpression caused by LPS alone, although this inhibition was significantly lower than that obtained in the presence of the *E. amentacea* SA extract.

## 3. Discussion

For the first time, the structural characterization of sodium alginate (SA) isolated from *E. amentacea* end-products, specifically from apices wasted by a cultivation step during an algal forest restoration, is reported. The extraction yield of the *E. amentacea* SA was about 3.05% (*w*/*w*), which is close to the 5.43% reported by Zrid and collaborators [32] for alginate extracted from another species of the same complex, *Cystoseira humilis*. This yield was lower by about three-fold than that obtained with other species of *Cystoseria* complex, such as *Gongolaria barbata* SA (9.9% [33]) and *Ericaria sedoides* (11% [34]) harvested from the Tunisian coasts. Moreover, the obtained yield was much lower compared with other species, such as *Cystoseira crinita* (20.18% [15]), *Cystoseira compressa* (21.65% [35]), *Laminaria digitate* (36% [36]), *Macrocystis pyrifera* (18–45% [37]), *Laminaria ochroleuca* (50% [38]), *Durvillaea antarctica* (53%), *Ecklonia cava* (35–38%) [39], *Durvillaea potatorum* (55% [40]). As demonstrated in Table 1, SA contained 44.25% total sugars and 49% uronic acids, with the presence of some impurities like proteins (0.53%) and phenolic compounds (0.93%). All of these results were in accordance with the literature concerning SA extracted from other algal species, such as *Padina perindusiata*, *G. barbata*, and *C. compressa* [33,35,41]. This broad disparity in the extraction yields and chemical composition of all of these brown seaweeds might be explained by some differences in the extraction procedures but also by the seasonal trends and ecological factors, as well as the species’ own characteristics [33,37]. In our case, the use of the seaweed apices after gamete release induction for the ex situ cultivation step for algal restoration purposes may also explain the lower SA yield from the present *E. amentacea* samples. Indeed, the seaweed apices could present lower SA content either because they are younger formations and/or because they need a different structural plasticity/flexibility, as compared to the stiffness of the mature thalli.

The calculated M/G ratio of the *E. amentacea* SA was 2.33, indicating that this polymer is primarily composed of 70% mannuronic acid (F_M_ = 0.7) and 30% guluronic acid (F_G_ = 0.3). Similar results have been reported in studies on other algal species. For instance, SA extracted from *D. potatorum*, *Saccorhiza polyschides*, and *D. antarctica* exhibited mannuronic/guluronic (M/G) ratios of 2.125, 2.14, and 2.15, respectively [38]. This ratio was lower than those reported in the literature for SA extracted from *Turbinaria turbinata* (M/G = 2.66), *Durvillaea willana* (M/G = 2.57), *Fucus guiryi* (M/G = 4.41), *Sargassum cinereum* (M/G = 4.5), and *Dictyota dichotoma* (M/G = 4.8) [29,38,42]. These values are considered high, as several algal SAs typically exhibit M/G ratios ranging between 0.45 and 1.86 [15]. According to the literature, M/G ratios are influenced by factors such as algal maturity, seasonal environmental conditions, and the extraction method used [11]. In addition, the various biological activities of SA polysaccharides are often closely related to their physical properties in aqueous media. The physicochemical characteristics of alginate depend not only on the M/G ratio but more fundamentally on the molar frequencies of uronic acid pairings—specifically, the homopolymeric block structures (F_MM_; F_GG_) and the alternating blocks (F_MG_/_GM_). These structural parameters significantly influence the chemical and physical behavior of alginates, as the stiffness of the polymer chain increases in the order MM-block < MG-block < GG-block. Therefore, this allows for the classification of alginates as high-M (F_M_ > 0.7), low-M (F_M_ < 0.6), and intermediate-M (F_M_, 0.6–0.7) [43]. Based on our results, the alginate extracted from the *E. amentacea* algal apices yielded an intermediate-M (F_M_ = 0.7), which contained a higher homopolymeric fraction (F_MM_ = 0.66; F_GG_ = 0.26) compared to heteropolymeric blocks (F_GM_/_MG_ = 0.04), with a predominance of MM-blocks (η = 0.190), allowing for more flexibility compared to seaweed thalli [44]. A complete description of the alginate monomer sequence is not possible only through diad analysis (F_GG_, F_MM_, F_GM_, and F_MG_), as described previously. Nevertheless, the η parameter, defined as η = F_MG_/(F_M_ × F_G_), can be used to predict the sequence distributions in algal alginates. Therefore, values of η < 1 indicate the abundance of MM and GG homopolymeric block types, whereas η = 1 reveals completely random cases, and 1 < η < 2 illustrates alternate-like cases of MG and GM [30,45]. The calculated η value for the extracted alginate was 0.190 (η < 1), indicating a polymer structure predominantly composed of homopolymeric block sequences.

Structural characteristics of SA might be tied to the functional and ecological roles of *E. amentacea* apices. In fact, *E. amentacea* is typically found in shallow, intertidal, or upper subtidal habitats, where it is constantly exposed to strong hydrodynamic forces such as waves, currents, and tidal fluctuations. In such environments, mechanical flexibility is critical to preventing structural damage. The apical regions are younger, actively growing tissues that must maintain structural integrity while still being pliable enough to withstand water movement. Higher MM content in alginate allows these tissues to bend and flex without breaking, acting almost like a shock absorber against mechanical stress. Moreover, the apices are also sites of cell division and elongation, requiring a less rigid and more hydrated cell wall to accommodate expansion [46,47]. Mannuronic acid-rich alginate forms softer, more elastic gels, which likely facilitate cell wall remodeling during growth and morphogenesis. Indeed, it is important to note that the composition and structure of alginate can vary along different parts of the algal body. The stipe or basal regions may contain more guluronic acid-rich alginate (a lower M/G ratio), which forms stiffer, more rigid gels (due to GG-block dominance), contributing to mechanical strength and anchorage. In contrast, the apex may maintain a mannuronic-rich composition specifically to preserve tissue flexibility, which is essential for both growth and hydrodynamic performance in exposed shallow-water habitats [15,48,49]. From a biotechnological perspective, the structural characteristics of SA are generally very useful for many bioengineering applications, particularly for the production of soft hydrogels and flexible membranes. The high mannuronic acid (M) content leads to the formation of softer, more elastic gels that can better accommodate dynamic environments, ideal for tissue engineering, wound healing, and drug delivery systems where mechanical adaptability, moisture retention, and biocompatibility are critical [50,51]. These hydrogels can closely mimic the natural extracellular matrix, supporting cell growth and migration [52,53].

The estimated molecular weight (M_w_) for our SA extract was remarkably low compared to those reported in previous investigations on alginates from fucales, such as *E. sedoides*, *C. compressa,* and *G. barbata*, which had M_w_ values of 1 × 10^5^, 1.4 × 10^5^, and 2 × 10^5^, respectively [33,34,35]. SA polydispersity index q was around 3.5, indicating a partially hydrolyzed alginate, probably a result of the extraction process [33]. The SA intrinsic viscosity (37 mL/g) was ten times lower compared to other *Cystoseiraceae* species, which were in the range of 283–860 mL/g [33]. These results may be related to our extraction process, which differs from the standard protocol usually used to purify sodium alginate (SA). We employed a hydrothermal–alkaline extraction, starting with a thermal treatment (2 h at 120 °C) typically used for fucoidan polysaccharides, followed by a precipitation with CaCl_2_ and alkaline treatment to recover the alginate. This approach is sustainable, as it allows for the simultaneous extraction of both fucoidan and alginate polymers from the by-products of ex situ algal cultivation for restoration purposes. Thus, our extract cannot be considered crude alginate due to partial degradation; however, it also cannot be strictly classified as an oligosaccharide since this is generally between 200 and 4000 Da [54]. Furthermore, we used the apical (new growth) parts of the algae for restoration, which may also explain the lower molecular weight, as younger tissues contain lower M_w_ alginate compared to older parts [15].

The antioxidant potential of seaweed extracts makes them promising candidates for nutritional, pharmacological, and medical applications [55]. For this reason, the antioxidant activity of *E. amentacea* SA extract was evaluated using three different in chemico assays: overall radical-scavenging activity, Fe^3+^ reducing power, and nitric oxide (NO) scavenging activity. The DPPH assay is widely used to assess the free radical-scavenging ability of compounds and is a reliable method for evaluating the reducing properties of antioxidant substances [56]. The SA polysaccharide demonstrated a concentration-dependent antiradical activity. At the highest concentration (1.25 mg/mL), it showed a scavenging potential exceeding 90%, while at the lowest (0.125 mg/mL), the activity remained slightly above 30%. This result is comparable to that reported by Hentati et al. [35], who studied the DPPH scavenging activity of SA extracted from *C. compressa* and found approximately 46% activity at 0.5 mg/mL. However, this is lower than the scavenging activity observed for SA extracted from *G. barbata*, which reached 74% at the same concentration [33]. Moreover, the *E. amentacea* SA extract showed an IC_50_ value of approximately 0.41 mg/mL, which is lower (more potent) than the alginate extracted from *E. sedoides* (IC_50_ = 2 mg/mL [34]) and also lower than that of *C. compressa*, which showed an IC_50_ of around 0.56 mg/mL [35]. In general, the DPPH radical-scavenging effect of antioxidant polysaccharides is closely related to their hydrogen-donating ability. The mechanism of DPPH scavenging has been associated with several antioxidant processes, including chain inhibition, prevention of continued hydrogen abstraction, hydrogen donation, and direct radical scavenging [57]. According to the literature, mannuronic acid units form β-(1→4) bonds, resulting in linear and flexible M-block segments. In contrast, guluronic acid units are connected by α-(1→4) bonds, introducing steric hindrance around the carboxyl groups, which leads to folded, rigid conformations in the G-block segments [10]. Based on this information, the high mannuronic acid content of the extracted SA from *E. amentacea* may show higher chain flexibility, thereby exposing more –OH and –COO^−^ groups in M-blocks and enhancing radical-scavenging activity. In addition, low-molecular-weight alginates obtained by heat treatment have been reported to exhibit higher antioxidant activity than non-treated alginates [58], which is consistent with the lower molecular weight observed in our SA extract. These findings suggest that this *E. amentacea* SA extract possesses promising antioxidant properties, making it a potential bioactive molecule for pharmaceutical and nutraceutical applications.

Following the evaluation of the overall antioxidant capacity of the SA polysaccharide, its specific scavenging activity against highly reactive oxygen species (ROS) was investigated. This approach aimed to identify which ROS might be most effectively influenced at the intracellular level by the pharmacological application of *E. amentacea* SA. This analysis is particularly relevant, as ROS are commonly produced during oxidative stress and are closely associated with both acute and chronic inflammatory conditions. The Fe^3+^-reducing power of the SA extract was assessed using the potassium ferricyanide method. The reducing power of SA increased with concentration, ranging from 0.067 to 0.535 mg/mL, and reached an absorbance (A_700_ nm) of 1.06 at 0.535 mg/mL. This result is consistent with findings reported by Hentati et al. [35], who observed an A_700_ of approximately 1.0 at 0.5 mg/mL of SA extracted from *C. compressa*. Additionally, the reducing power observed in our study was higher than that reported by Sellimi et al. [33], who measured an A_700_ of 0.7 at the same concentration of SA extracted from *G. barbata*. These findings demonstrate that the *E. amentacea* SA can act as an effective electron donor, reacting with free radicals to convert them into more stable, non-reactive species and thereby interrupting radical chain reactions.

Additionally, the nitric oxide (NO) scavenging activity of the SA polysaccharide was evaluated. NO radicals are known to cause significant molecular damage both intracellularly and extracellularly under conditions of oxidative stress. Moreover, they function as secondary messengers in key signal transduction pathways, contributing to the propagation of the inflammatory response [59]. The extracted SA exhibited an in chemico NO-scavenging activity of 50% at a concentration of 0.417 mg/mL. Although no prior studies were found in the literature evaluating NO-scavenging activity specifically for SA, similar activities were reported for ethanol and DMSO extracts of *E. amentacea*, with calculated IC_50_ values of 0.546 mg/mL and 1.293 mg/mL, respectively [60]. However, in that case, the effect was mostly due to the presence of polyphenols and meroditerpenes species in those extracts, in the absence of polysaccharides. Notably, also in in vitro experiments, in the LPS-stimulated RAW 264.7 cellular model, after only 8 h, more than 40% inhibition of NO release in the extracellular medium was observed in the presence of the SA extract and maintained over time (i.e., up to 24 h). However, this inhibitory activity was not concentration-dependent, reaching a maximum at 0.2 mg/mL SA and not increasing at higher amounts. Moreover, the in vitro cellular tests also showed a pronounced inhibition of LPS-induced iNOS gene overexpression in RAW 264.7 murine macrophages, responsible for the massive production of NO during the inflammatory response, with over 70% suppression at both 8 and 24 h incubation times in the presence of SA. Conversely, by using two different commercial SAs characterized by low and medium molecular weights (CL and CM), we could not obtain the same remarkable inhibition of both NO production and iNOS expression. In fact, only a slight effect for the two commercial SA was observed (~20% NO release at 24 h and iNOS overexpression at 8 and 24 h, respectively). These different results could indicate that the inhibitory effect on this important inflammatory pathway could be due to the specific chemical composition of each SA and/or to the different molecular weight and tridimensional conformation of the various SA polymers. These effects are particularly noteworthy since, in addition to exerting direct antioxidant activity by scavenging NO excess produced by this enzyme, *E. amentacea* SA can also inhibit the upstream expression of iNOS at the mRNA level, thereby reducing both its transcription and protein production. This dual mechanism has the potential to effectively diminish the damage caused by excessive NO. These results are in accordance with those reported for some alginate oligosaccharides [61,62], indicating a similar effect compared to our low-M_w_ SA hydrothermal extract. Overall, these data demonstrate the very promising activity of the *E. amentacea* SA, which could pave the way for the use of this seaweed-derived product as a tool to reduce inflammatory foci, where the NO overproduction often leads to significant tissue damage if not counteracted by pharmacological intervention [63].

Inflammation plays a key role in the development of several pathological conditions, including cancer, obesity, and cardiovascular diseases [64,65]. Toll-like receptors (TLRs), particularly TLR4, are central to the molecular mechanisms underlying these conditions [66]. When activated by lipopolysaccharide (LPS), TLR4 triggers signaling pathways that lead to the activation of the transcription factor NF-κB, as well as several kinases, such as Akt, MAPK, and PI3K. This cascade results in the increased synthesis of inflammation-related mediators, including prostaglandin E2 (PGE_2_) and nitric oxide (NO), produced by the pro-inflammatory enzymes COX-2 and iNOS, respectively, and pro-inflammatory cytokines [67,68]. As a consequence, excessive or chronic inflammation may have detrimental effects on the body. In this context, polymers such as alginate have been widely studied for their anti-inflammatory properties, owing to their high biocompatibility [69,70,71]. In addition to its notable antioxidant and NO-scavenging activity, SA extracted from *E. amentacea* demonstrated significant anti-inflammatory effects. These effects were observed in both RAW 264.7 murine macrophages and differentiated human THP-1 M0 macrophages following LPS treatment for 8 and 24 h. Notably, in both cell lines, the SA extract at 24 h significantly reduced the expression levels of key pro-inflammatory cytokines, including IL-6, IL-8/CXCL5, and TNF-α, which are crucial mediators in propagating inflammatory signals and recruiting immune cells to the site of inflammation. The anti-inflammatory potential of our SA extract was especially evident in human THP-1 M0 macrophages, where the suppression effect of IL-6, IL-8, MCP-1, and TNF-α was even more prominent than in murine macrophages.

Conversely, by using the same dose of SA for a shorter incubation time in the presence of LPS (8 h), we observed inhibitory effects only on some inflammatory mediators in RAW 264.7 murine macrophages. Specifically, the expression levels of IL-6, CXCL5 (i.e., the functional homolog of human IL-8 in mice), and iNOS were significantly suppressed, whereas IL-1β and TNF-α expression levels showed an increase, while COX-2 remained unaffected by the presence of *E. amentacea* SA. Thus, there is a limitation in the anti-inflammatory potential of the new SA extract, which is indeed absent for LPS-induced IL1-β and COX-2, where no efficacy could be claimed. At the same time, parallel experiments performed on commercial CL and CM SA showed the very low anti-inflammatory potential of these two polymers as compared to the *E. amentacea* SA extract. The inhibitory effect was only observed by use of the medium-molecular-weight SA (CM) versus IL-6, IL-8, TNF-α, and MCP-1 cytokine overexpression, indicating different degrees of efficacy of the various SA obtained from marine organisms. Our results indicate that this inhibitory potential likely depends both on the different monomer chemical composition, as well as on the average molecular weight of the polysaccharide extracted, since our SA shows a significantly lower M_w_ as compared to the commercial ones. Notably, all SAs tested in this study failed to exert any inhibition of COX-2 overexpression and also caused a significant IL-1β increase in the presence of LPS early stimulation (8 h) in murine macrophages, indicating that these polysaccharides may act at different levels of the various cytokine induction pathways, with different results for each cytokine. Furthermore, the two commercial SAs caused a significant increase in IL-1β and IL-6 at 24 h in LPS-stimulated murine macrophages, and these results are partially comparable to those reported by Yang and Jones [72], who demonstrated that SA stimulated RAW 264.7 macrophages, increasing the levels of pro-inflammatory cytokines IL-1β, IL-6, IL-12, and TNF-α and activating innate immune responses through NF-κB signaling. This pro-inflammatory effect was attributed to an SA containing high proportions of M- and MG-blocks. Similar observations have been reported in other studies [15]. While these findings partially align with our results, we observed a contrasting outcome, in that *E. amentacea* SA at the same incubation time significantly inhibited the expression of IL-6, CXCL5, and iNOS, and even more at longer incubation times. Indeed, at 24 h of incubation, where most cytokines were significantly inhibited by the *E. amentacea* SA, both in murine and human macrophages, our findings are in contrast with previous reports that suggest that high mannuronic acid content in SA enhances its immunogenicity and promotes immunostimulatory effects [73,74]. In fact, the SA used in this study, composed of 70% mannuronic acid and 30% guluronic acid, exhibited significant anti-inflammatory and antioxidant activities in two macrophage cellular models. Since our *E. amentacea* SA showed a lower average molecular weight than other alginates, we may at least partially attribute the anti-inflammatory effect exerted on immune cells to this difference, other than the presence of other components in the SA extracts from other marine organisms, as compared to the one we obtained through a hydrothermal process from this brown seaweed. Notably, it is frequently reported that brown algae alginate extracts retain low levels of phenolic compounds [34,35,42,75,76] and that achieving completely phenolic-free alginate from brown seaweed is technically challenging [42]. Thus, although sodium alginate represents the largely predominant component of the *E. amentacea* extract and is likely to play a major role in the biological effects observed, a partial contribution of residual polyphenols cannot be excluded.

These in vitro results for *E. amentacea* SA in immune cells also demonstrate promising therapeutic potential for in vivo systems, where dampening these signals is essential to extinguishing inflammatory foci and reducing the possibility of the chronicization of the process since all the above-mentioned cytokines are involved in feeding and propagating the inflammation at the tissue level [77]. Notably, the ability of the SA extract to inhibit a wide range of critical inflammatory mediators—including cytokines and NO synthase—suggests that its mechanism of action may occur upstream in the inflammatory signaling cascade. This could involve interference with key signals, like an intracellular ROS increase, for example, which is known to activate transduction pathways that lead to macrophage activation and subsequent changes in gene expression profiles during the inflammatory response [78]. Similar to our findings in vitro, other authors have reported the anti-inflammatory effects of alginate in in vivo studies. Indeed, various forms of alginate, from oligosaccharides to microspheres or hydrogels, have already been used to alleviate ulcerative colitis and joint inflammatory disorder symptoms with promising lenitive and anti-inflammatory outcomes [63,79,80,81]. Furthermore, alginate has been shown to exert local anti-inflammatory effects, and it is already in use for rectal administration in the treatment of chronic hemorrhoids, proctosigmoiditis, and chronic anal fissures following surgical interventions in the rectal region [82]. Additionally, alginate has been reported to promote mucous membrane regeneration, suppress gastric inflammation, and support the restoration of intestinal microbial flora. These polymers have also demonstrated beneficial effects in treating inflammation of the gastric and esophageal mucosa, as well as radiation-induced stomatitis [83]. Notably, the SA extracted from *E. amentacea* would add the remarkable NO-scavenging effect and inhibition of iNOS production to the already known properties of polysaccharides, increasing the pharmacological success of alginate therapies in all the above-mentioned diseases. Thus, many possibilities for employment seem to lie ahead for this SA extract obtained from *E. amentacea* Mediterranean brown seaweed.

## 4. Materials and Methods

### 4.1. Chemicals

Ethanol, CaCl_2_ Na_2_CO_3_, HCl, NaOH, phenol, H_2_SO_4_, glucose, uronic acid, carbazole, Bradford reagent, Folin–Ciocalteu reagent, D2O, 2,2-diphenyl-1-picrylhydrazyl (DPPH), ascorbic acid, Na_2_HPO_4_, potassium ferricyanide, trichloroacetic acid, FeCl_3_, sodium nitroprusside, Griess reagent, NaNO_2_, 3-(4,5-dimethyl-2-yl)-2,5-diphenyltetrazolium bromide (MTT), lipopolysaccharide (LPS), SDS, DTT, glycerol, and TRIS-HCl, alginic acid low viscosity, and alginic acid medium viscosity were purchased from Sigma-Aldrich (Milan, Italy). DMEM medium, fetal bovine serum (FBS), and RPMI 1640 medium were acquired from Microtech srl (Naples, Italy). Phorbol-12-myristate 13-acetate (PMA) was purchased from PeproTech EC (London, UK). RNeasy Mini Kit was obtained from Qiagen (Milan, Italy). cDNA Synthesis Kit and 4× CAPITAL™ qPCR Green Master Mix were acquired from biotechrabbit GmbH (Henningsdorf, Germany).

### 4.2. Raw Material

In the Ligurian Sea (Northwestern Mediterranean), apices of *Ericaria amentacea* were harvested in the midlittoral zone, on exposed rocky shores, in Bogliasco, Genoa (NW Italy, 44 2°2′40.37″ N–9°4035.14″ E), in the summer of 2023. After retrieval, the apices were stored in plastic bags kept in cold conditions and immediately transported to the DISTAV laboratory at the University of Genoa. The biomass was thoroughly washed with sterilized seawater to remove sand particles and epiphytes. In the culture facility, the apices were used to seed restoration units following the protocol by Falace et al. [28]. After the release of gametes, the waste biomass from the ex situ culture step was dried at 55 °C for 22 h in the dark to prevent any possible degradation associated with light. The dried sample material was ground using a blender, passed through a 0.5 mm sieve, and kept in plastic bags at −20 °C until use.

### 4.3. Sodium Alginate Extraction

**Figure 8:**
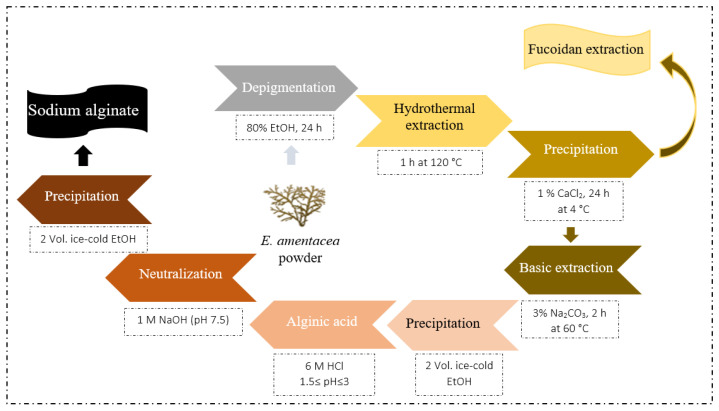
Processing steps for the extraction of alginate from *E. amentacea* dry seaweed biomass.

*E. amentacea* sodium alginate was extracted by hydrothermal–alkaline extraction [35,84] with some editing (Figure 8). Initially, 10 g of dried apex seaweed powder underwent mild depigmentation and delipidation in EtOH (80% *v*/*v*, 24 h). Afterward, the residue was subjected to a thermal treatment at 120 °C for 1 h in an autoclave (Vapor matrc 770, Asal srl, Milan, Italy). The extraction was performed in duplicate, and solids were separated from the liquid by centrifugation (3040 rpm, 13 min). A solution of 35% CaCl_2_ was added to the supernatant, corresponding to 1% CaCl_2_ final concentration, and kept at 4 °C overnight. Subsequently, the wet pellet obtained after centrifugation was treated with 100 mL of Na_2_CO_3_ (3% (*w*/*v*), pH = 11) at 60 °C for 2 h under stirring (the supernatant was stocked at −20 °C for fucoidan extraction). After centrifugation at 4000 rpm for 25 min at 4 °C, the supernatant was treated with 2 volumes of 96% ice-cold ethanol (−20 °C) for 15 min, with slight manual stirring, for polysaccharides precipitation. The precipitate was separated by centrifugation (4000 rpm, 45 min, 4 °C), resolubilized in deionized water, and then acidified with 6 M HCl to precipitate the alginic acid at a pH value of 1.5 ≤ pH ≤ 3. The pellet formed after centrifugation (4000 rpm, 30 min, 4 °C) was resuspended in 40 mL of deionized water and neutralized (pH = 7.5) with an aqueous NaOH (1 M) solution. The obtained sodium alginate (SA) was finally precipitated with two volumes of ice-cold ethanol (96%) for 15 min, and the resulting suspension was centrifuged at 4000 rpm for 45 min at 4 °C. Finally, the polysaccharide pellet was solubilized (50 g/L) and lyophilized to obtain the SA. The yield was calculated using the equation below:

Yield of SA (%) = (weight of SA/weight of seaweed dried biomass) ×100

### 4.4. Physicochemical Characterization

#### 4.4.1. Centesimal Composition

Total sugars in SA were measured by the phenol-H_2_SO_4_ method [85], using glucose as a standard. Meanwhile, uronic acids were determined using the carbazole method [86] and glucuronic acid as a standard. The quantification of protein and polyphenol contents was accomplished using the Bradford [87] and Folin–Ciocalteu methods [88], respectively.

#### 4.4.2. Spectral Analyses

- Fourier-transform infrared spectroscopy

The Fourier-transform infrared (FTIR) spectra of the SA were analyzed to identify the possible functional groups as a variation in functional groups in the standard, using a Bruker Vertex 70 spectrometer (Bruker, Milan, Italy). The spectrum was recorded over the range 4000–400 cm^−1^ with 128 scans at a resolution of 2 cm^−1^ by using the platinum ATR: a single reflection diamond ATR sampling accessory.

- NMR spectroscopy

SA was dissolved in D_2_O (99.9% D) at a concentration of 6 g/L. After dissolution, the sample was freeze-dried, resulting in sodium alginate with exchangeable protons replaced by deuterium. In total, this step was performed 3 times. Before analysis, the SA-lyophilized sample was dissolved again in D_2_O (99.9% D) at a concentration of 8.5 g/L. NMR spectra were obtained at 80 °C using a Jeol JNM-ECZ400R (Akishima, Tokyo, Japan) (400 MHz for ^1^H) with a Royal HFX probe. All NMR spectra were analyzed using the software MestReNova (Mestrelab Research^®^ v. 14.2) (Santiago de Compostela, Spain). A spectral width of 4000 Hz was used to acquire the data obtained under the following acquisition parameters: acquisition mode = 2 s, pulse 90° = 8 µsec, scans = 64, recovery = 5 s (for a complete return after the 90° pulse).

#### 4.4.3. Molecular Weight, Viscosity, and Polydispersity Determination

The molecular weight of extracted alginate was determined using intrinsic viscosity [η] measurements obtained via a capillary viscometer. The viscosimetric average molecular weight, M_v_, was estimated using the Mark–Houwink–Sakurada equation, which relates the intrinsic viscosity to the molecular weight of the polymer:

[η] = kM_v_^α^

where k (cm^3^/g) and α are parameters obtained from the literature and depend on the polymer–solvent system and temperature.

A known amount of polymer was dissolved in a 0.1 mol/L NaCl solution, as most of the Mark–Houwink parameters reported in the literature for alginate were determined using measurements in this aqueous medium to prevent polyelectrolyte expansion in solution.

For measurements at 20 °C, with alginate in solution of 0.1 mol/L NaCl, K assumes a value of 4.85 × 10^−3^ cm^3^/g and α of 0.97 [89].

The weight average molecular mass, M_w_, and the number average molecular mass, Mn, were then calculated by means of empirical equations with constants reported in [90], and the polydispersity index q (M_w_/Mn) was determined.

### 4.5. Biological Activities

#### 4.5.1. Antioxidant Activity

- DPPH radical-scavenging activity

The DPPH (2,2-diphenyl-1-picrylhydrazyl) radical-scavenging capacity of SA was evaluated by Kirby and Schmidt’s method [91]. Briefly, 500 μL of the samples at different concentrations (from 0.125 to 1.25 mg/mL) was mixed with ethanol (99%, 375 μL) and a DPPH solution in ethanol (125 μL, 0.02% (*w*/*v*)). The solution was incubated for 30 min at room temperature in the dark. The absorbance was read at λ = 517 nm using a NanoDrop One/Onec spectrophotometer (ThermoFisher, Milan, Italy). Ascorbic acid was used as a positive standard, and then, the DPPH scavenging activity was estimated following the equation below:

DPPH radical-scavenging activity %= ((A_control_ + A_blank_) − A_sample_)/Acontrol × 100

where A_control_, A_blank_, and A_sample_ are, respectively, the absorbance of the control reaction (absence of sample), the blank (without DPPH solution), and the SA extracts in the DPPH solution. The procedure was carried out in duplicate.

- Reducing Fe (III) Power Assay

The ability of sodium alginate to reduce iron (III) was determined using the method reported by Sampath Kumar et al. [92]. SA dilutions (0.067–0.535 mg/mL) were mixed with 250 μL of 200 mM phosphate buffer (pH 6.6) and 250 μL of 1% potassium ferricyanide. The mixture was incubated at 50 °C for 30 min, and then, 250 μL of 10% (*w*/*v*) trichloroacetic acid was added. The mixture was then centrifuged at 10,000 rpm for 10 min. In total, 500 μL of the supernatant was mixed with 500 μL of deionized water and 100 μL of 0.1% (*w*/*v*) FeCl_3_. After 10 min, the absorbance was measured at λ = 700 nm using a NanoDrop One/Onec spectrophotometer. The procedure was performed in duplicate. The negative control was produced by replacing the samples with water, while the positive control consisted of ascorbic acid. Results were expressed as absorbance of Fe^2+^ at 700 nm at different concentrations.

- NO-Scavenging Activity

NO-scavenging activity was determined using the assay reported by Xu et al. [93]. Various dilutions of SA (from 0.042 to 0.417 mg/mL) in 250 µL of phosphate buffer (0.1 M, pH 7.4) were added to 500 µL of 5 mM sodium nitroprusside and 250 µL of phosphate buffer (pH 7.4). To produce NO, samples were incubated under the light of a lamp at room temperature for 30 min. Afterward, an equal volume of Griess reagent was added to the mixture. After incubation at room temperature for 10 min in the dark, the absorbance was measured at 546 nm through a NanoDrop One/Onec spectrophotometer. For the calibration curve, NaNO_2_ scalar dilutions were used (1–5–10–50 µM).

#### 4.5.2. Anti-Inflammatory Activity

- Cell culture

The murine RAW 264.7 cell line and the human THP-1 cell line were acquired from the American Type Culture Collection (LGC Standards srl, Milan, Italy). RAW 264.7 cells were cultured in high-glucose D-MEM with L-glutamine, supplemented with 10% FBS (Microtech srl, Naples, Italy). THP-1 cells were maintained in RPMI 1640 supplemented with 10% FBS (Microtech). Cell incubation was carried out in 5% CO_2_ atmosphere, 95% humidity, and 37 °C temperature for both cell types.

- Cytotoxicity assay

Cell viability was assessed with the MTT (3-(4,5-dimethyl-2-yl)-2,5- diphenyltetrazolium bromide) reduction assay, as reported in Pozzolini et al. [90]. Seeding of RAW 264.7 macrophages was carried out at a density of 2.5 × 10^4^ cells/well in 96-well plates. THP-1 monocytes were seeded at a density of 5 × 10^4^ cells/well in 96-well plates, and differentiation into M0 macrophages was performed by treatment with 20 ng/mL of phorbol myristate acetate (PMA) for 48 h. Both cell lines were treated by adding SA at different concentrations (0.05, 0.1, 0.2, and 0.4 mg/mL) for 24 h at 37 °C. Experiments were performed in quintuplicate.

- NO-Scavenging in LPS-Treated RAW 264.7 Macrophages

RAW 264.7 macrophages were seeded at 1 × 10^6^ cells/well in 6-well plates for 24 h. Cells were then challenged with bacterial lipopolysaccharide (LPS, 100 ng/mL) in the presence or absence of SA (0.2 and 0.4 mg/mL) or low and medium viscosity alginic acid (CL and CM, respectively, 0.4 mg/mL) for 8 and 24 h. Afterward, the nitrite content of the cell media was measured by the Griess assay [94], while cells were lysed in 300 µL of lysis buffer (2% SDS, 100 mM DTT, 10% glycerol, and 50 mM TRIS-HCl, adjusted to pH 6.8). The lysates were heated at 100 °C for 10 min, and the quantification of protein concentration was performed by the Bradford assay [87]. Nitrite production in each sample was then calculated using a standard NaNO_2_ curve and normalized on the protein content.

- Gene expression analysis of inflammatory mediators

RAW 264.7 macrophages were plated at a density of 1 × 10^6^ cells/ well in 6-well plates. Instead, THP-1 monocytes were seeded at a density of 5 × 10^5^ cells/well in 6-well plates and differentiated into M0 macrophages by treatment with 20 ng/mL PMA for 48 h. Both cell types were challenged with LPS (100 ng/mL) in the presence or absence of SA (0.2 and 0.4 mg/mL for RAW 264.7 macrophages and 0.4 mg/mL for THP-1 M0 cells), or of CL or CM at 0.4 mg/mL for both cell lines, for 8 and 24 h. At the end of each incubation, the RNA was extracted using the RNeasy Mini Kit (Qiagen, Milan, Italy), according to the manufacturer’s instructions. Quality and quantity of RNA were analyzed using a NanoDrop One/Onec spectrophotometer. The cDNA was synthesized from 1 μg of RNA by using a cDNA Synthesis Kit (biotechrabbit GmbH, Henningsdorf, Germany). Gene expression of the inflammatory mediators interleukin 1 β(IL-1β), interleukin-6 (IL-6), interleukin-8 (IL-8), tumor necrosis factor (TNF-α), cyclooxygenase-2 (COX-2), and monocyte chemoattractant protein-1 (MCP-1) was quantified by qPCR for THP-1 M0 cells. Moreover, the gene expressions of IL-1β, IL-6, TNF-α, COX-2, inducible NO synthase (iNOS), and C-X-C Motif Chemokine Ligand 5 (CXCL5) were also quantified in RAW 264.7 macrophages. Gene expression values were normalized on HPRT-1 and GAPDH housekeeping genes for THP-1 M0 cells and RAW 264.7 macrophages, respectively.

Each qPCR reaction was performed in 10 μL of 4× master mix (biotechrabbit GmbH), 0.2 µM of each primer, and 5 ng of cDNA. All samples were analyzed in triplicate. The following thermal conditions were used: initial denaturation at 95 °C for 3 min, followed by 45 cycles with denaturation at 95 °C for 15 s, and annealing and elongation at 60 °C for 60 s. The fluorescence was measured at the end of each elongation step. All primers (Table 4) were designed using the Beacon Designer 7.0 software (Premier Biosoft International, Palo Alto, CA, USA) and obtained from TibMolBiol (Genova, Italy). Data analyses were obtained using the DNA Engine Opticon 3 Real-Time Detection System Software program (3.03 version), and in order to calculate the relative gene expression compared to an untreated (control) calibrator sample, the comparative threshold Ct method was used within the Gene Expression Analysis for iCycler iQ Real Time Detection System software v3.1 (Bio-Rad, Milan, Italy).

### 4.6. Statistical Analysis

Statistical analyses were performed using one-way ANOVA and a paired Tukey’s post-test to assess significant differences (GraphPad Software, Inc., San Diego, CA, USA). * *p* <0.05 and ** *p* < 0.01 were considered significant.

## 5. Conclusions

Sodium alginate (SA) isolated from seaweed apices discarded from an ex situ cultivation procedure during the restoration of *Ericaria amentacea* in the Ligurian Sea was characterized using spectroscopic and intrinsic viscosity methods, including FTIR and ^1^H NMR. These marine polymers were characterized by low molecular weight compared to other alginates and exhibited notable antioxidant properties, as demonstrated by various chemical assays. Additionally, SA showed significant anti-inflammatory activity by modulating LPS-induced inflammatory mediators, including IL-6, IL-8/CXCL5, MCP-1, and TNF-α gene expression, in both human THP-1 M0 macrophages and RAW 264.7 murine macrophages. Furthermore, we showed the remarkable NO radical-scavenging capacity and a significant gene expression inhibition of iNOS, the enzyme responsible for its production in macrophages, suggesting possible pharmacological use of *E. amentacea* SA in counteracting the damaging effects of exacerbated inflammation at the tissue level. These findings highlight the potential of *E. amentacea* polysaccharides, especially when sustainably sourced from restoration procedure waste, for future industrial and biomedical applications.

## Figures and Tables

**Table 1:** Chemical content of *E. amentacea* SA.

Sample	Yield (%)	Total Sugar (%)	Uronic Acid (%)	Total Polyphenols (%)	Protein (%)
SA	3 ± 0.78	44.25 ± 4	49 ± 2.2	0.93 ± 0.08	0.52 ± 0.04

**Table 2:** Structural characterization of SA extracted from *E. amentacea* harvested in the Ligurian Sea.

Fraction	F_G_^1^	F_M_^2^	F_GG_^3^	F_GM_ or F_MG_^4^	F_MM_^5^	M/G ^6^	η ^7^
SA	0.30	0.70	0.26	0.04	0.66	2.33	0.160

^1^ F_G_—fraction of individual blocks of guluronic acid units; ^2^ F_M_—fraction of individual blocks of mannuronic acid units; ^3^ F_GG_—fraction of homogeneous block of guluronic acid; ^4^ F_GM_ or F_MG_—fraction of heterogeneous blocks of alternating mannuronic and guluronic acids; ^5^ F_MM_—fraction of homogeneous block of mannuronic acid; ^6^ M/G—ratio between F_M_ and F_G_; ^7^ η—parameter defined as η = F_MG_/(F_M_ × F_G_).

**Table 3:** Molecular weight, number average molecular weight, polydispersity index, and intrinsic viscosity of SA extracted from *E. amentacea* harvested in the Ligurian Sea.

Sample	M_W_ (g/mol)	M_n_ (g/mol)	q (M_w_/M_n_)	[η] (mL/g)
SA	1 × 10^4^	2.9 × 10^3^	3.5	36.8

**Table 4:** List of primer pairs used in the qPCR analyses. h- indicates human genes, m- indicates murine genes.

Gene	GenBank (a.n.)	Forward	Reverse	Product Size (bp)
h-IL-1β	NM_000576.3	TgATggCTTATTACAGTggCAATg	gTAgTggTggTCggAgATTCg	140
h-IL-6	NM_001318095.2	CAgATTTgAggTAgTgAggAAC	CgCAgAATgAgATgAgTTgTC	194
h-IL-8	NM_000584.4	AATTCATTCTCTgTggTATC	CCAggAATCTTgTATTgC	127
h-MCP-1	NM_002982	CTTCTgTgCCTgCTgCTC	CTTgCTgCTggTgATTCTTC	156
h-TNF-α	NM_000594.4	gTgAggAggACgAACATC	gAGCCAgAAgAggTTgAg	113
h-HPRT-1	NM_000194.3	ggTCAggCAgTATAATCCAAAg	TTCATTATAgTCAAgggCATATCC	144
m-IL-1β	NM_008361.4	gCAgCACATCAACAAgAg	CAgCAggTTATCATCATCATC	184
m-IL-6	NM_031168.2	ACCTgTCTATACCACTTC	gCATCATCgTTgTTCATA	117
m-CXCL5	NM_009141.3	TgCTTAACCgTAACTCCAA	ATCCAgACAgACCTCCTT	129
m-iNOS	NM_010927.4	CCgCCgCTCTAATACTTA	TTCATCAAggAATTATACAggAA	121
m-TNF-α	NM_013693.3	CCACCATCAAggACTCAA	ATCTTATCCAgCCTCATTCT	120
m-COX-2	NM_011198.5	CCAgCAAAgCCTAgAgCAAC	AgCACAAAACCAggATCAgg	127
m-GAPDH	NM_001289726.1	TCTCCCTCACAATTTCCATCCCAg	gggTgCAGCgAACTT TATTgATgg	99

## Data Availability

All data are reported within this manuscript.

## Supplementary Materials

The following supporting information can be downloaded at https://www.mdpi.com/article/10.3390/md24010041/s1: Figure S1: Commercial acid alginic L and M (CL and CM respectively) inhibition of gene expression in LPS-activated RAW 264.7 macrophages.; Figure S2: Commercial acid alginic L and M (CL and CM respectively) inhibition of gene expression in THP-1 M0 macrophages.; Figure S3: NO scavenging activity and iNOS gene expression in cellular assay.

## Abbreviations

The following abbreviations are used in this manuscript:

ANOVA	Analysis of variance
COX-2	Cyclooxygenase-2
CXCL5	C-X-C Motif Chemokine Ligand 5
DNA	Deoxyribonucleic acid
DPPH	2,2-diphenyl-1-picrylhydrazyl
FTIR	Fourier-transform infrared
GAPDH	Glyceraldehyde 3-phosphate dehydrogenase
HPRT-1	Hypoxanthine-guanine phosphoribosyltransferase 1
IL-1β	Interleukin 1 beta
IL-6	Interleukin 6
IL-8	Interleukin 8
iNOS	Inducible nitric oxide synthase
LPS	Lipopolysaccharide
MCP-1	Monocyte chemoattractant protein-1
MTT	3-(4,5-dimethyl-2-yl)-2,5- diphenyltetrazolium bromide
NMR	Nuclear Magnetic Resonance
NO	Nitric oxide
PMA	Phorbol myristate acetate
qPCR	Quantitative polymerase chain reaction
RNA	Ribonucleic acid
ROS	Reactive oxygen species
SA	Sodium alginate
TNF-α	Tumor necrosis factor alpha
UV	Ultraviolet
