# Supplementary material for: Structural Characterization and Anti-Inflammatory Properties of an Alginate Extracted from the Brown Seaweed Ericaria amentacea
Source: Mar Drugs. 2026 Jan 13;24(1):41. doi: 10.3390/md24010041 (PMC12842944; doi:10.3390/md24010041)
Supplement: Supplementary file 1 [file marinedrugs-24-00041-s001.zip › marinedrugs-3985804-supplementary.pdf]

marine drugs

Structural Characterization and Anti-inflammatory Properties of an Alginate Extracted from the Brown Seaweed *Ericaria amentacea*

Maha Moussa [1,2], Serena Mirata [1,3], Lisa Moni [4], Valentina Asnaghi [1,2], Marina Alloisio [4], Simone Pettineo [4], Maila Castellano [4], Silvia Vicini [4], Mariachiara Chiantore [1,2] and Sonia Scarfi [1,2,3]

[1] Department of Earth, Environment and Life Sciences, University of Genoa, Italy; maha.moussa@edu.unige.it (M.M); serenamira94@gmail.com (S.M); valentina.asnaghi@unige.it (V.A); mariachiara.chiantore@unige.it (M.C); sonia.scarfi@unige.it (S.S)
[2] National Biodiversity Future Center (NBFC), 90133 Palermo, Italy; maha.moussa@edu.unige.it (M.M); valentina.asnaghi@unige.it (V.A); mariachiara.chiantore@unige.it (M.C); sonia.scarfi@unige.it (S.S)
[3] Inter-University Centre for the Promotion of the 3Rs Principles in Teaching & Research, 10129, Turin, Italy; serenamira94@gmail.com (S.M); sonia.scarfi@unige.it (S.S)
[4] Department of Chemistry and Industrial Chemistry (DCCI), University of Genoa, Italy; Lisa.Moni@unige.it (L.M); Marina.Alloisio@unige.it (M.A); pettineo.simone@gmail.com (S.P); Maila.Castellano@unige.it (M.CA); Silvia.Vicini@unige.it (S.V)
* Correspondence: sonia.scarfi@unige.it ; Tel.: +393402894173

 https://doi.org/10.3390/md24010041

a

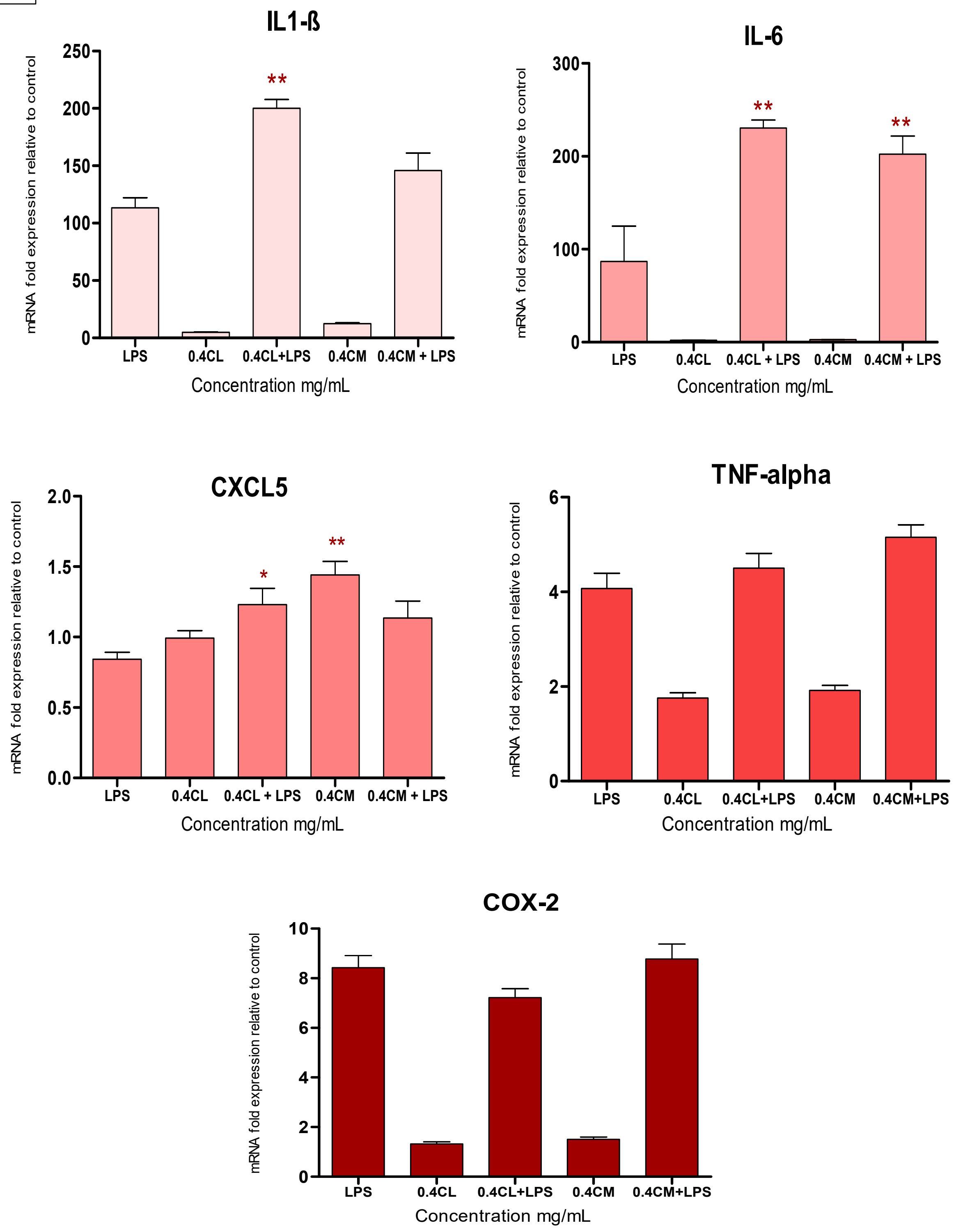

b

https://doi.org/10.3390/md24010041

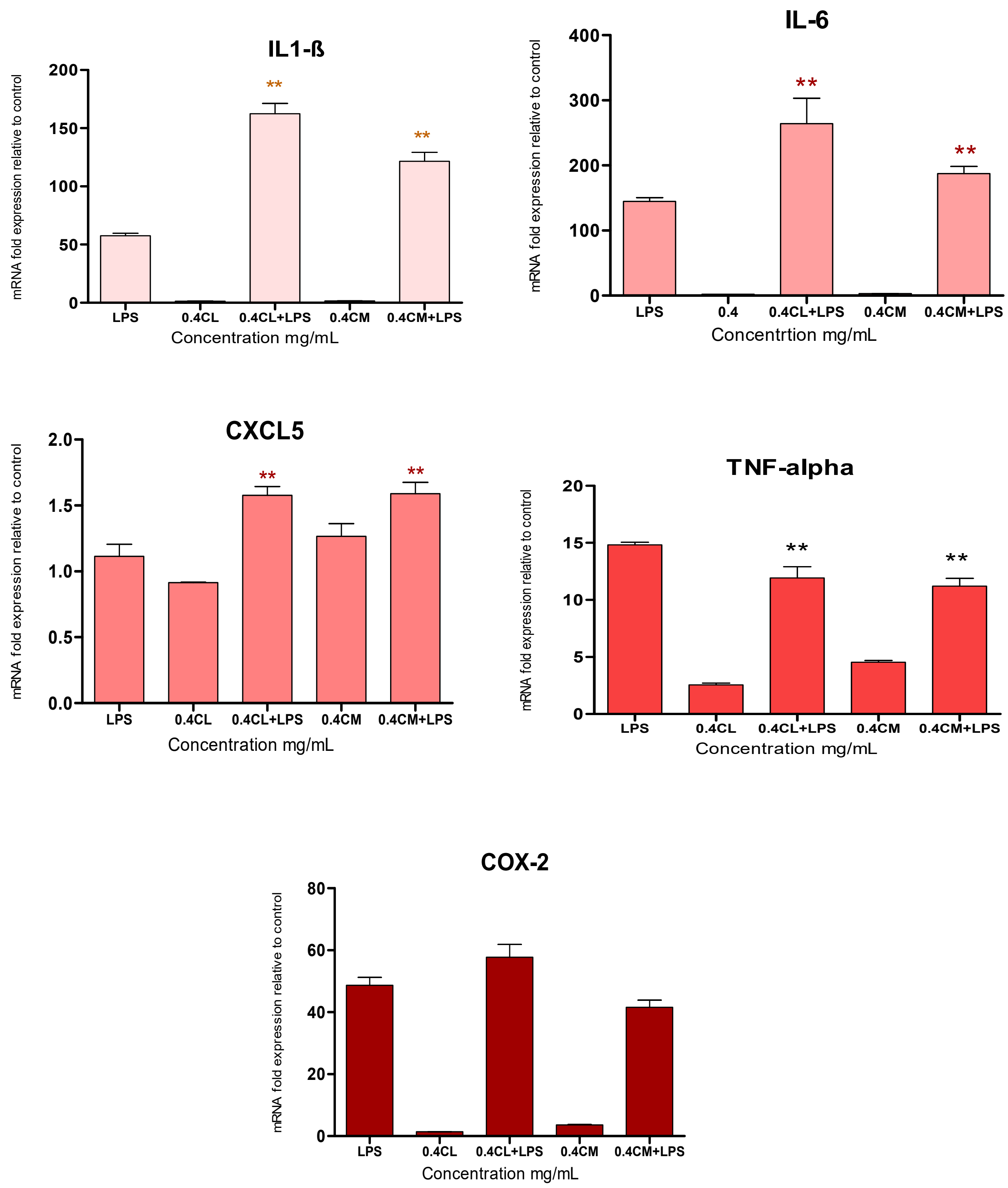

Figure S1. Commercial acid alginic L and M (CL and CM respectively) inhibition of gene expression in LPS-activated RAW 264.7 macrophages. Gene expression measured by qPCR analysis of IL-1β, IL-6, CXCL5, TNF-α, and COX-2, after incubation for 8 h (**a**) and 24 h (**b**), with or without LPS (100 ng/mL), and in presence or absence of 0.4 mg/mL CL and CM. Data are normalized on the GAPDH housekeeping gene and expressed as mRNA fold increase compared to control, untreated cells. Results are the mean ± SD of three experiments performed in duplicate. ANOVA was significant in each histogram ($p < 0.05$), with * $p < 0.05$, and ** $p < 0.01$ vs LPS in Tukey post-test.

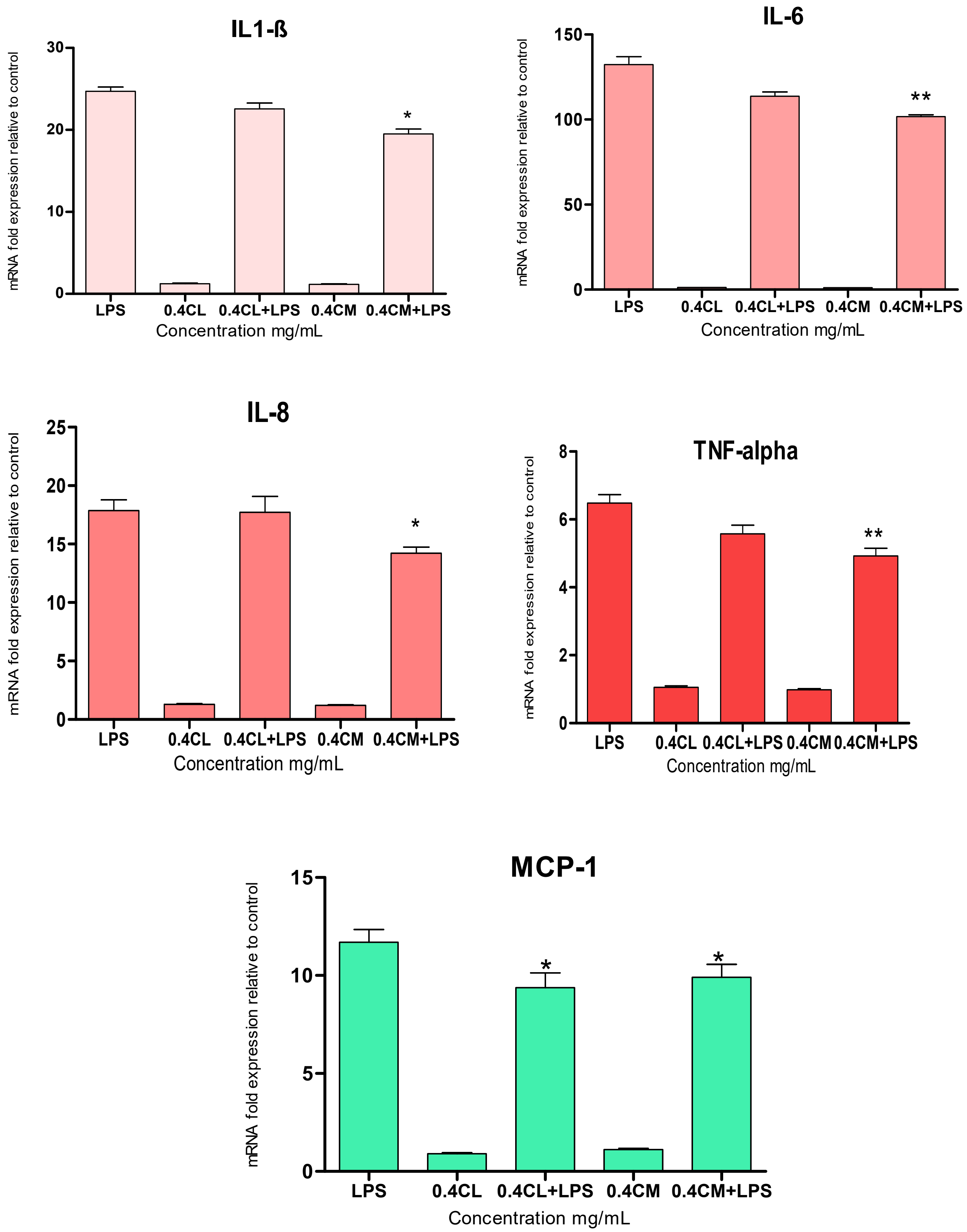

Figure S2. Commercial acid alginic L and M (CL and CM respectively) inhibition of gene expression in THP-1 M0 macrophages. Gene expression measured by qPCR analysis of IL-1β, IL-6, IL-8, TNF-α and, MCP-1 after THP-1 M0 macrophages incubation, with or without LPS (100 ng/mL) and in presence or absence of 0.4 mg/mL CL and CM for 24 h. Data are normalized on the HPRT-1

https://doi.org/10.3390/md24010041

housekeeping gene and expressed as mRNA fold increase compared to control, untreated cells. Results are the mean ± SD of three experiments performed in duplicate. ANOVA was significant in each histogram ($p < 0.05$), with * $p < 0.05$, and ** $p < 0.01$ vs LPS in Tukey post-test.

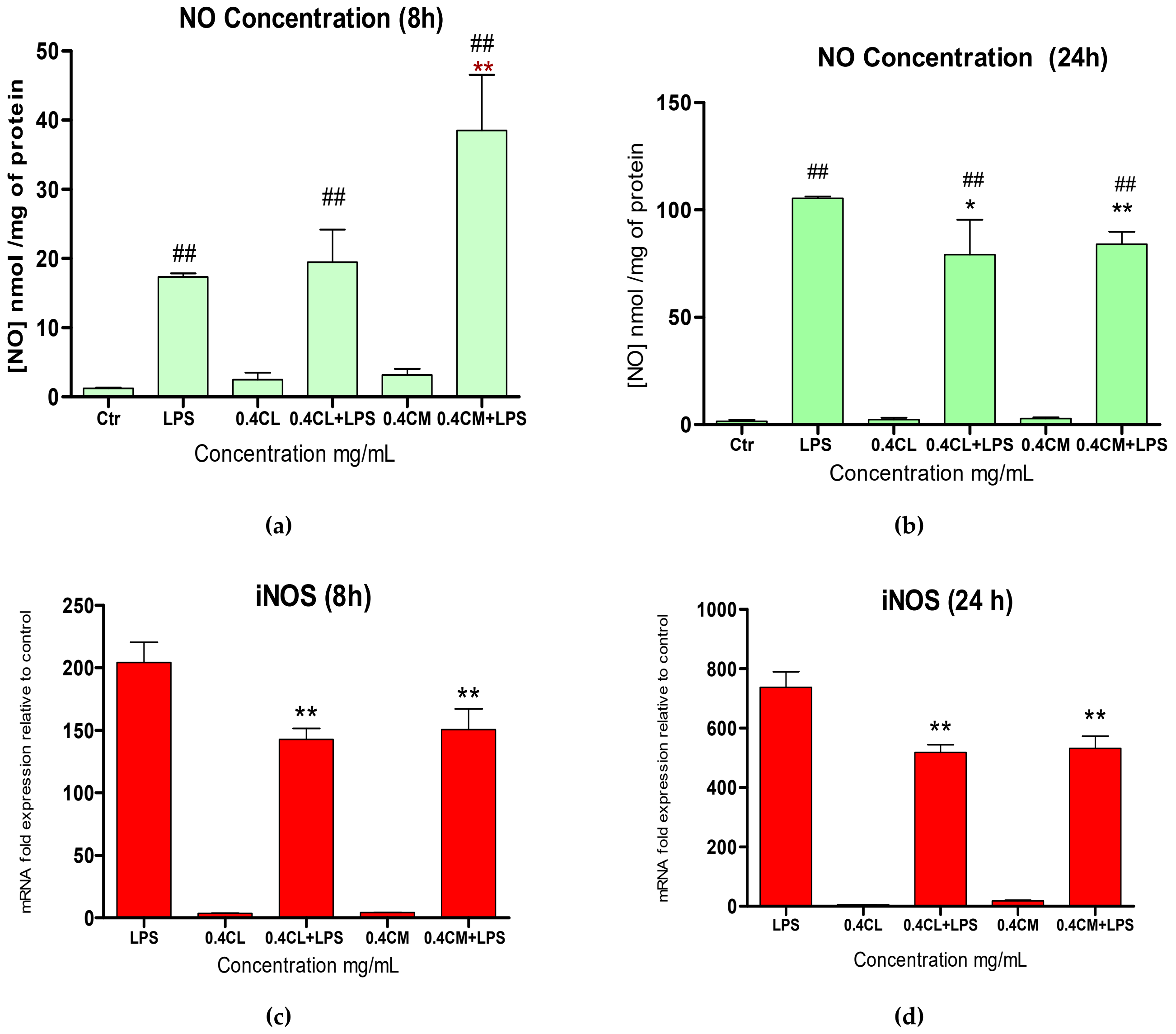

Figure S3. NO scavenging activity and iNOS gene expression in cellular assay. (a, b) Intracellular NO production measured by Griess assay in RAW 264.7 murine macrophages incubated for 8 h (a) and 24 h (b) with LPS (100 ng/mL) in presence or absence of 0.4 mg/mL CL and CM. The results are expressed as percentage of NO production respect to control untreated cells and are the mean ± SD of two assays performed in triplicate. (c, d) Gene expression measured by qPCR analysis of iNOS after RAW 264.7 cell incubation, for 8 h (c) and 24 h (d), with or without 100 ng/mL LPS and in presence or absence of 0.4 mg/mL CL and CM. Data are normalized on the GAPDH housekeeping gene and expressed as mRNA fold increase compared to control, untreated cells. Results are the mean ± SD of three experiments performed in duplicate. ANOVA was significant in each histogram ($p < 0.05$), with * $p < 0.05$, and ** $p < 0.01$ vs LPS in Tukey post-test; and $^{\#}p < 0.05$, and $^{\#\#}p < 0.01$, vs negative control in Tukey post-test.

https://doi.org/10.3390/md24010041
